# *Priestia megaterium* ASC-1 Isolated from Pickled Cabbage Ameliorates Hyperuricemia by Degrading Uric Acid in Rats

**DOI:** 10.3390/microorganisms12040832

**Published:** 2024-04-20

**Authors:** Wenjuan Zhu, Siyuan Bi, Zhijia Fang, Lukman Iddrisu, Qi Deng, Lijun Sun, Ravi Gooneratne

**Affiliations:** 1 Guangdong Provincial Key Laboratory of Aquatic Product Processing and Safety, Guangdong Provincial Engineering Technology, Research Center of Marine Food, Key Laboratory of Advanced Processing of Aquatic Products of Guangdong Higher Education Institution, College of Food Science and Technology, Guangdong Ocean University, Zhanjiang 524088, China; 2112103084@stu.gdou.edu.cn (W.Z.); bisiyuan0129@126.com (S.B.); lukmaniddrisu54@gmail.com (L.I.); dengqi@gdou.edu.cn (Q.D.); suncamt@126.com (L.S.); 2 Department of Wine, Food and Molecular Biosciences, Lincoln University, Lincoln 7647, Canterbury, New Zealand; ravi.gooneratne@lincoln.ac.nz

**Keywords:** hyperuricemia, uric acid, *Priestia megaterium*, gut microbiota

## Abstract

Pickled cabbage, a traditional fermented food rich in functional microorganisms, can effectively control hyperuricemia and gout. In this study, a *Priestia megaterium* ASC-1 strain with strong uric acid (UA) degradation ability was isolated from pickled cabbage. After oral administration for 15 days, ASC-1 was stably colonized in the rats in this study. ASC-1 significantly reduced UA levels (67.24%) in hyperuricemic rats. Additionally, ASC-1 alleviated hyperuricemia-related inflammatory response, oxidative stress, and blood urea nitrogen. Intestinal microbial diversity results showed that ASC-1 restored intestinal injury and gut flora dysbiosis caused by hyperuricemia. These findings suggest that *P. megaterium* ASC-1 may be used as a therapeutic adjuvant for the treatment of hyperuricemia.

## 1. Introduction

Uric acid (UA) is a heterocyclic compound with poor water solubility and produced during purine metabolism [1]. Due to the absence of genes encoding uricase in humans, excessive intake of purine or decreased excretion of UA can result in high levels of UA in the blood, leading to hyperuricemia [2], as well as the emergence of medical disorders such as hypertension, gout, kidney disease, and elevated vulnerability to cardiovascular disease [3,4]. The prevalence rates of hyperuricemia vary by region, with the highest incidence rates observed in maritime countries [5,6]. Currently, hyperuricemia is attracting widespread attention.

The prevention and treatment of hyperuricemia remain urgent issues. To date, the primary principle for managing hyperuricemia has been to obstruct uric acid production and enhance uric acid excretion [7,8]. Nevertheless, prolonged use of uric acid-lowering medicines may cause adverse effects, such as serious allergic reactions and renal impairment [8]. In recent years, scientists have explored the mitigating effects of microbes on hyperuricemia with the goal of creating safer and more effective medications [9,10]. Several microorganisms that are effective in lowering uric acid levels, including *Lactobacillus gasseri* (PA-3), *Lactobacillus rhamnosus* (Fmb14), and *Lactobacillus fermentum* (JL-3), have been reported [11,12,13]. These microorganisms can reduce hyperuricemia by regulating the intestinal flora and affecting metabolic processes. The risk of hyperuricemia is elevated due to microbiota imbalance [6,14,15,16]. Thus, gut microorganisms and their balance are critical to the prevention and treatment of hyperuricemia.

Research has shown that the microorganisms in fermented foods can effectively maintain the balance of the intestinal flora, regulate the immune system, improve intestinal inflammatory diseases, and promote nutrient absorption [17,18,19,20,21]. Furthermore, microorganisms isolated from fermented foods have been shown to have a significant impact in combating a range of health issues, especially hyperlipidemia [22,23,24,25,26,27,28]. Studies have shown that the traditional fermented food pickled cabbage is rich in functional microorganisms and can effectively control hyperuricemia and gout. The microorganisms in pickled cabbage have been found to lower the levels of nitrite and sulfide and degrade purine [29,30,31,32]. Thus, the present study aimed to screen microbial strains from pickled cabbage with UA degradation ability and to evaluate the effects of these strains on a high-purine diet and oxonic acid potassium-induced hyperuricemia in a rat model [33].

## 2. Materials and Methods

### 2.1. Isolation of the Bacterial Strain with UA-Degrading Ability

Samples of pickled cabbage were procured from the Zhanjiang Marketplace located in Zhanjiang, Guangdong province, China. A total of 376 bacterial strains were isolated from these samples using UA medium, which consists of 1.71 g of Na_2_HPO_4_·12H_2_O, 0.3 g of KH_2_PO_4_, 0.05 g of NaCl, 0.05 g of MgSO_4_·7H_2_O, 0.001 g of CaCl_2_, 0.5 g of UA, and 1.2 g of agar per 100 milliliters of medium. The isolation process was conducted at 37 °C as previously described [11]. The isolated strains were then cultured on UA-based medium, which served as the sole carbon source. Subsequently, the bacterial colonies (ASC-1, S-1, and S-2) were streaked and purified on new nutrient agar plates to obtain single colonies. Newly obtained single colonies were preserved in 50% glycerol at −80 °C.

### 2.2. Determination of Uric Acid Degradation Ability of Strains In Vitro

The inosine degradation ability of the strains was evaluated as previously described [33], and an activated bacterial solution was promptly introduced into a fresh nutrient agar medium at a volume of 1%. After inoculation, 2 mL of the culture medium was centrifuged at 4 °C and 4 commissions r/min for 10 min, confidently. The organisms were collected and washed twice with sterile 0.9% NaCl solution. After resuspending the organisms, they were incubated at 37 °C and 120 r/min for 1 h. Following centrifugation, the supernatant was added into a 0.1 mol/L HClO_4_ solution at a ratio of 9:1 by volume with complete confidence. To halt the reaction, 0.1 mol/L of HClO_4_ was added without any second thoughts. The supernatant was filtered using a microporous membrane, and the inosine content was analyzed using HPLC with complete assurance. The degrading speed and rate of inosine by different strains were calculated according to Formulas (1) and (2):

V = (0.9 C − X)/60(1)

D = [(0.9 C − X)/0.9 C] × 100%(2)

where V is the degrading speed (g/L/min), X is the remaining content of inosine (g/L), and D is the degradation rate. Inosine-neutral potassium dihydrogen phosphate culture medium (900 μL) was combined with 100 μL of perchloric acid solution to terminate the reaction. Then, 20 μL of the resulting mixture was aspirated and injected into a high-performance liquid chromatography system (HPLC, Agilent 1200, Santa Clara, CA, USA). The retention time of inosine was accurately determined using an external standard method, and a standard curve was generated for quantitative analysis. The HPLC conditions were carefully optimized and standardized, using a Symmetry C18 column (4.6 mm × 250 mm) with a mobile phase consisting of water and chromatographic methanol in a volume ratio of 95:5. The flow rate was maintained at 0.7 mL/min, while the column temperature was set at 25 °C. The detection wavelength was chosen to be 254 nm, corresponding to the absorption maximum of inosine, and the elution time was fixed at 14 min to ensure complete separation and accurate quantification.

### 2.3. 16S rRNA Gene Sequencing and Phylogenetic Analysis

For strain identification, pure cultured strains were subjected to streak purification. The 1492S rRNA sequence was amplified using the primers 16F (AGAGTTT-GATCCTGGCTCAG) and 27R (GGTTACCTTGTTACGACTT). Subsequently, the polymerase chain reaction (PCR) protocol was standardized. The resulting amplification products were analyzed by 1% agarose gel electrophoresis, enabling the visualization of DNA fragments using a gel imaging system (Sangon Biotech, Shanghai, China). Finally, the PCR products were sent to Bioengineering Co. (Shanghai, China) for sequencing. Isolates were identified using the BLAST algorithm in the EZBioCloud database (https://www.ezbiocloud.net/). The morphology of ASC-1 was analyzed by scanning electron microscopy (JSM-7610F thermal field emission scanning electron microscope, JEOL (Beijing) Co., Ltd., Beijing, China), while multiple sequences of ASC-1 and other closely related strains were aligned using MEGA (Molecular Evolutionary Genetics Analysis, v.6.0).

### 2.4. Experimental Design Based on the Animal Model

Forty SD rats (age: 4 weeks; weight: 200–230 g) were purchased from Guangzhou Kexuan Biotechnology Co., Ltd. (Guangzhou, China), and habituated for one week before the experiment. Each rat was housed individually in a cage maintained at a temperature of 22 ± 1 °C and a relative humidity of 60−75%. Subsequently, the rats were administered a synergistic maintenance diet. The ethical treatment and utilization of experimental animals in this study were strictly approved by the Animal Ethics Committee of Guangdong Ocean University (permission number: GDOU-LAE-2023-032). Strict adherence to relevant ethical standards was maintained throughout the experiment.

Under specific pathogen-free conditions, male SD rats were randomly assigned to four groups (*n* = 10 in each group): a control group given a standard diet, normal drinking water, and sterile saline (1 mL) administrated by oral gavage every day (control); a UA group given a high-purine diet, normal drinking water, intraperitoneal injection of potassium oxalate, and orally gavaged sterile saline (1 mL) every day (UA); a treatment group gavaged with *B. megaterium* ASC-1 at a dose of 1 × 10^9^ CFU/kg/day and given a high-purine diet and intraperitoneal injection of potassium oxazinate for 2 weeks (UA + ASC-1); and rats in a positive control group were orally gavaged with allopurinol (30 mg/kg) daily, fed a high-purine diet, and injected intraperitoneally with potassium oxonate for 2 weeks (allopurinol).

At the end of the study, feces and urine samples from each rat were collected and stored at −80 °C until further analysis. After fasting for 12 h, the rats were anesthetized with 10% chloral hydrate and euthanized by carotid bleeding. Tissue samples were excised from the liver, kidney, and intestine and meticulously rinsed with sterile phosphate-buffered saline. The samples were promptly frozen at −80 °C for further analysis. Serum was obtained by centrifuging the blood samples at 1500× *g* for 10 min. The resulting serum was stored at −80 °C until further analysis. Owing to variations in the indicators among samples, it is crucial to meticulously choose a specific number of samples for analysis.

### 2.5. Colonization of Priestia Megaterium ASC-1

During the course of the experiment, fecal samples were systematically collected from rats on days 4, 8, and 12. PCR was then performed using specific primers for *Bacillus megaterium* as reported in previous literature [34]. All primer sequences for qPCR were as follows: F: TGCTAGAGCTTCATTTAGGTATGGC; R: TAGAACTGTTTTAGCATCACGAGAC. Then, a semi-quantitative fluorescence analysis of the PCR products of ASC-1 was performed using the ViiA™ 7 Dx [35].

To quantify the total bacteria in the fecal samples, we weighed 50 mg of fecal sample (25th day) in a 2 mL EP tube, put the tube on ice, and added 500 μL of buffer SA, 100 μL of buffer SC, 15 μL of Proteinase K, 0.25 g of grinding beads, and ground with a tissue grinder to mix the sample thoroughly. Subsequently, it was extracted in strict accordance with the instructions of the “TIANamp Stool DNA Kit”, and the final elution volume was 50 μL. All measurements were performed in triplicates. Recombinant plasmids were used as calibration standards for quantitative polymerase chain reaction (qPCR) analysis. These plasmids were serially diluted to obtain 6 × 10^4^, 6 × 10^5^, 6 × 10^6^, 6 × 10^7^, and 6 × 10^8^ copies per reaction. Subsequently, the target copy numbers (T) were estimated using the equation T = LOG (quality/copies, 10). At least five 10-fold dilutions of the plasmids were performed in triplicate, and standard curves were generated from these data points. Finally, the proportion of ASC-1 within the entire bacteria community in various samples was calculated based on these standard curves.

### 2.6. Determination of UA in Rats

An appropriate amount of frozen rat feces was collected, added proportionally to the PBS solution, and then dissolved in a 60 °C water bath. The sample was vortexed for 1 min and centrifuged at 1000 rpm for 5 min. The supernatant was then collected for further analysis. Frozen rat urine samples were thawed in a 60 °C water bath and diluted with distilled water until they were free of urate crystals. All samples were assayed according to the instructions of the uric acid assay kit supplied by Jiancheng (Nanjing, China).

### 2.7. Determination of Short-Chain Fatty Acids (SCFAs) in Rat Feces

After collecting the samples, they were sent to Qingdao Future Testing-Technology Co., Ltd. (Qingdao, China) for the detection of short-chain fatty acids. Subsequently, an appropriate number of samples and 1 mL of water were added and mixed well, and then 300 μL of 50% sulfuric acid was added, followed by 100 μL of 500 mg/L internal standard (cyclohexanone) solution and 2 mL of ether. The mixture was homogenized for 1 min and centrifuged at 12,000 rpm at 4 °C for 10 min. The supernatant was used for testing. A chromatographic system was used for analysis. The Agilent DB-WAX capillary column (30 m × 0.25 mm × 0.25 μm) was used with high-purity helium (purity not less than 99.999%) as the carrier gas at a flow rate of 1.0 mL/min. The inlet temperature was set to 220 °C, and a non-split injection of 1 μL was used with a solvent delay time of 2.5 min. The mass spectrometry system employed an electron impact ion source (EI) with an ion source temperature of 230 °C and an interface temperature of 220 °C. Integration was performed using the GCMS Solution software (Shimadzu GCMS QP2010-Ultra, Kyoto, Japan), and the content was calculated using the standard curve.

### 2.8. Tissues Collection and Biomarker Measurements

Fresh liver and kidney tissues were homogenized at 4 °C using a high-speed tissue grinder to obtain sample suspensions. Subsequently, the suspension was centrifuged at a low speed of 8000 rpm for 10 min. Commercial kits (Grace Biotechnology, Suzhou, China) were used in accordance with the manufacturer’s instructions. Xanthine oxidase (XOD) and interleukin-1β (IL-1β) were assayed in liver samples, while malondialdehyde (MDA) and IL-1β levels were measured in kidney samples. Serum samples were analyzed for IL-1β, MDA, creatinine (CRE), and urea nitrogen (BUN). At least five samples per group were selected for independent determination.

Liver and kidney samples were washed with PBS. The liver volume was 0.8 cm × 0.8 cm, while the entire kidney was used. The samples were fixed with a 4% paraformaldehyde solution at room temperature for more than a day. Subsequently, the samples were sent to Wuhan Saiweier Biotechnology Co., Ltd. (Wuhan, China) for physiological sectioning and hematoxylin and eosin (HE) staining. The renal tubules of different groups of rats were observed under a microscope to examine the morphology of the sections and accumulation of UA.

### 2.9. Degradation Ability of UA by Rats’ Fecal Flora

Fecal samples were collected from rats at various time points. Following the protocol outlined above, 1 g of feces was cultured in YCFA medium and incubated under anaerobic conditions at 37 °C for 48 h. The samples were then transferred to an incubator and incubated for two days. The optical density was measured at 600 nm (OD600). Subsequently, the medium was centrifuged at 8000× *g* for 10 min using low-temperature high-speed centrifugation to isolate the bacterial cells. The obtained bacterial bodies were washed twice with sterile phosphate-buffered saline (PBS). After washing, the bacteria were resuspended in pure PBS containing uric acid (UA). The supernatant from the incubator was collected every three days for further analysis. The changes in UA concentration were detected using a UA detection kit (Jiancheng, Nanjing, China) according to the manufacturer’s instructions.

### 2.10. DNA Extraction and 16S rRNA Gene Amplification

Upon completion of sample collection, the samples were sent to Guangzhou Gene Denovo Biotechnology Co., Ltd. (Guangzhou, China) for intestinal flora detection. After the extraction of genomic DNA from the sample, the V3 + V4 regions of the 16S rDNA (16S rRNA gene) were amplified using specific barcoded primers. The primer sequences were as follows: 341F: CCTACGGGNGGCWGCAG; 806R: GGACTACHVGGGTATCTAAT. The purified amplification products (i.e., amplicons) were then coupled to sequencing adapters to construct a sequencing library for Illumina sequencing.

### 2.11. Statistical Analysis

Statistical analysis was conducted using GraphPad Prism version 8.0.1 software. A *p* value less than 0.05 was considered statistically significant. Data were analyzed using one-way analysis of variance (ANOVA). Independent sample *t*-tests were used to analyze significant differences between the two groups.

## 3. Results

### 3.1. Isolation of UA-Degrading Strains and Product Identification

**Figure 1:**
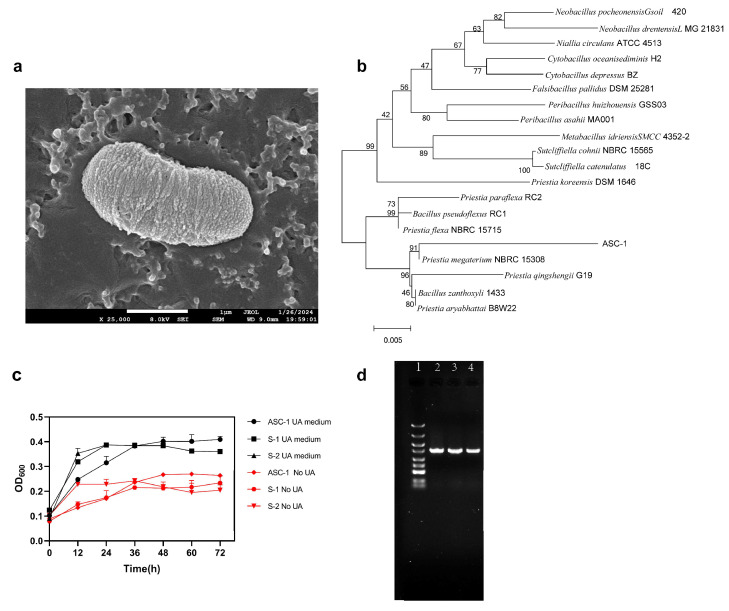
The screening and identification of ASC-1 strain. (**a**) Scanning electron microscopy (SEM) images of the ASC-1 strain. (**b**) Construction of a phylogenetic tree of the ASC-1 strain and related bacterial species. (**c**) The influence of different culture media on the growth of strains ASC-1, S-1, and S-2. Compared with the medium without uric acid, the three strains grew better in the medium containing UA, and ASC-1 had the best growth effect in the medium containing UA. (**d**) Agarose gel electrophoresis for the identification of isolated strains. Line 1: Marker DL5000; lines 2–4: 16S rDNA amplified from strains ASC-1, S-1, and S-2.

To investigate the potential benefits of the probiotics in pickled cabbage for hyperuricemia, we isolated 376 strains with a high level of uric acid tolerance from pickled cabbage. These strains were then cultured in a medium where uric acid was the sole source of carbon and nitrogen. Three strains—ASC-1, S-1, and S-2—showed the strongest growth ability in the given environment, and ASC-1 grew at a higher rate than the other two strains, with an OD600 value of more than 0.25 during the 24 h incubation period (Figure 1c). In comparison with the NCBI BLAST database, the ASC-1 strain was found to be closely related to *Priestia megaterium* NBRC 15308 (Figure 1b) [36]. Scanning electron microscopy (SEM) revealed that ASC-1 exhibited the typical morphological characteristics of *Bacillus Cohn* (Figure 1a).

**Figure 2:**
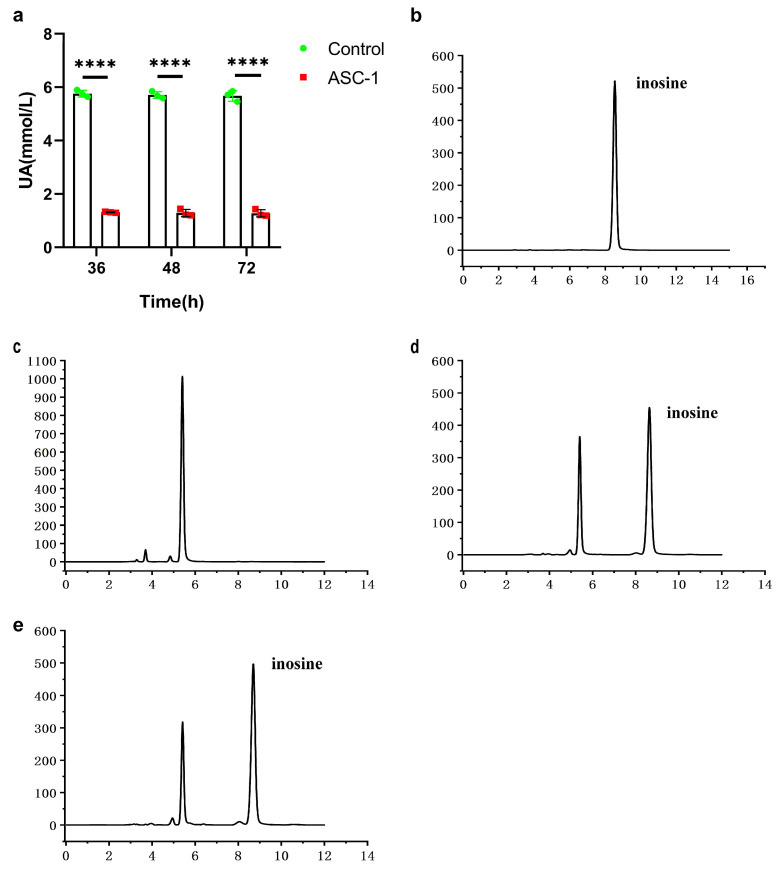
Detection of uric acid degradation products by ASC-1 strain. (**a**) The ability of ASC-1 to degrade UA in vitro. (**b**) Detection peak of inosine standard. (**c**) Detection of inosine degradation products by ASC-1 using high-performance liquid chromatography (HPLC). (**d**) Detection of inosine degradation products by S-1. (**e**) Detection of inosine degradation products by S-2. The bar graph shows the mean ± standard deviation (*n* = 3 samples per group). **** *p* < 0.0001, indicating statistically significant differences.

To analyze the ability of the ASC-1 strain to degrade uric acid (UA), ASC-1 was cultured in a sterile phosphate-buffered solution containing 6 mmol/L of UA. The results showed that the ASC-1 strain was able to degrade 77.14% of the total uric acid within 36 h, exhibiting higher degradation efficiency than the control (Figure 2a). In addition, the results of high-performance liquid chromatography (HPLC) showed absorption peaks at 5.407 and 8.639, assigned to the inosine and its degradation products, which were consistent with the standard peaks of inosine (Figure 2b). These results indicate that ASC-1 can more effectively decompose inosine into smaller molecules than S-1 and S-2 (Figure 2c–e).

### 3.2. ASC-1 Decreases UA Level in Hyperuricemic Rats

**Figure 3:**
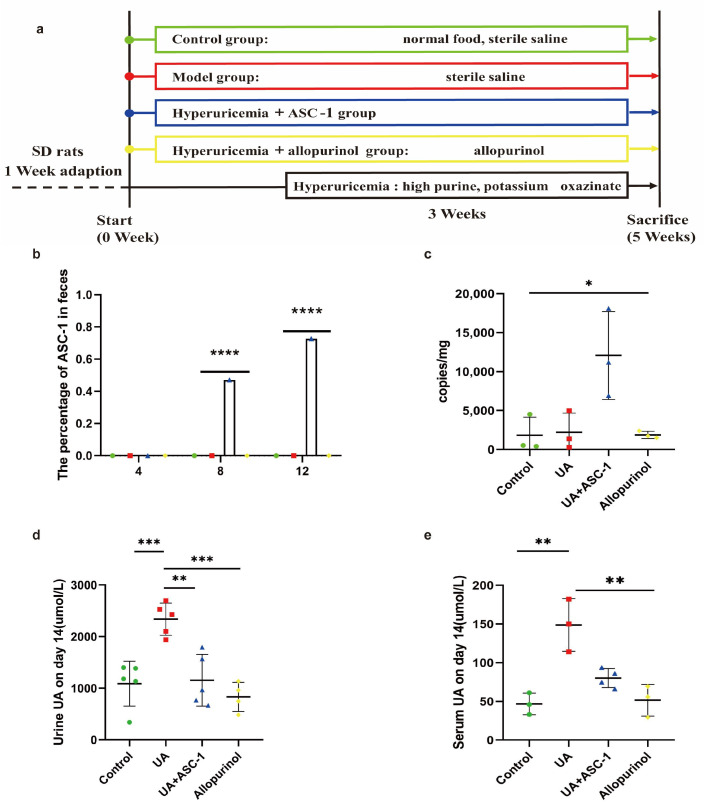
Effect of ASC-1 on hyperuricemia rats. (**a**) Flow chart of the experimental treatment of hyperuricemia rats with ASC-1. (**b**) Colonization of ASC-1 was detected by PCR on days 4, 8, and 12. (**c**) The proportion of ASC-1 in fecal flora was determined by qPCR on day 12. (**d**) The amount of UA in urine after 14 days. (**e**) Serum UA of rats after 14 days. Bar chart shows mean ± SD (*n* = 3 rats per group) * *p* < 0.1; ** *p* < 0.01, *** *p* < 0.001, **** *p* < 0.0001.

Previous research has confirmed that ASC-1 is capable of degrading UA and inosine. We subsequently established a hyperuricemic rat model and orally administered the hyperuricemic rats with the ASC-1 (Figure 3b). Fecal samples were collected to assess ASC-1’s survival in rats. By day 8, qPCR results showed a significantly higher relative abundance of ASC-1 in the feces of rats receiving the supplement compared to controls (Figure 3c). On day 12, the calculated amount of ASC-1 reached to 1.20 × 10^4^ copies/mg feces.

Further analysis indicated that ASC-1 colonization resulted in reduced uric acid levels in both the blood and urine samples of treated rats compared to those in the uric acid group. By day 15, serum samples indicated that the uric acid level in the uric acid group was 249.9 micromoles per liter, about 3.17 times higher than that of the control group at 148.86 micromoles per liter. After intervention with ASC-1 and allopurinol, there was a significant decrease of approximately 67.24% and 95.29% in uric acid concentration (*p* < 0.01), respectively, compared to the uric acid group, as shown in Figure 3e. It is important to note that there were no significant differences in uric acid levels between the ASC-1 group, allopurinol, and the control groups in urine and blood samples, indicating that ASC-1 effectively reduced uric acid levels in hyperuricemic rats.

### 3.3. ASC-1 Strain Attenuated UA-Induced Oxidative Stress and Inflammation

**Figure 4:**
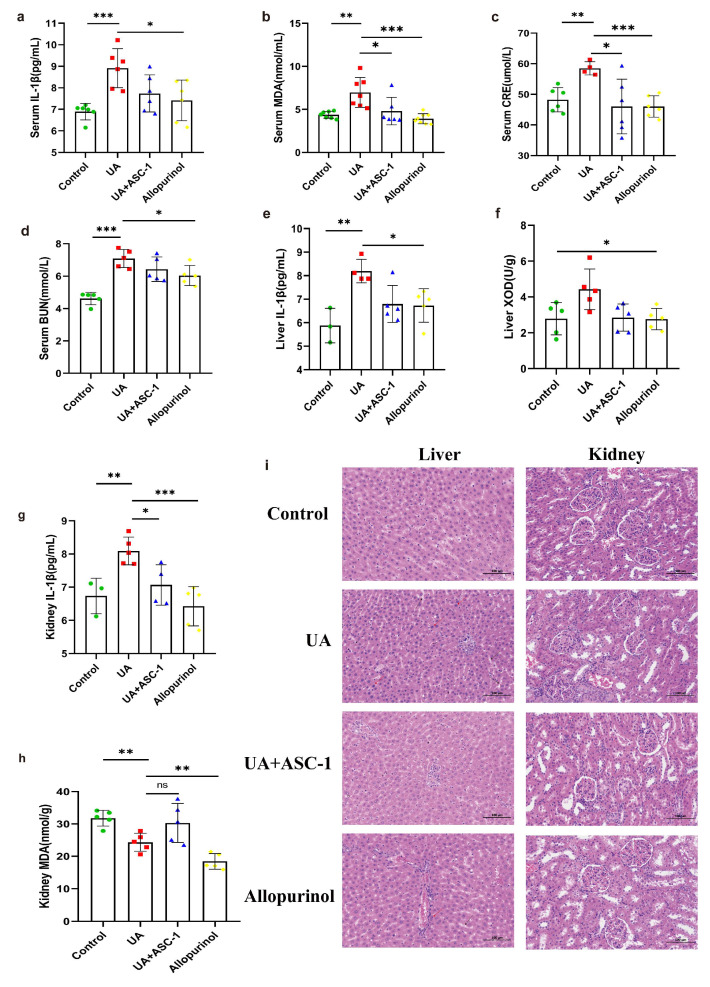
Effects of ASC-1 on inflammatory markers and oxidative stress indicators in hyperuricemia rats. (**a**–**d**) The levels of IL-1β, MDA, CRE, and BUN of rats from each group. (**e**–**h**) The levels of IL-1β and MDA in livers and kidneys of rats from each group. (**i**) Representative micrographs of liver and kidney tissues stained with hematoxylin and eosin (H&E). The bar graph displays the mean ± standard deviation for 5 rats in each group. Statistical significance was observed with * *p* < 0.1; ** *p* < 0.01; *** *p* < 0.001, and ns *p* > 0.05. The microscope magnification is 200 × Bar = 100 μm.

To investigate the physiological impact of ASC-1 in rats, we analyzed various biochemical markers. Specifically, we measured IL-1β, MDA, CRE, and BUN levels. The rats in the UA group showed an upward trend in these indicators (*p* < 0.01), as depicted in Figure 4a–d. Furthermore, the levels of these biomarkers were significantly reduced in the serum of rats from the ASC-1 + UA and allopurinol groups compared to those in the UA group (*p* < 0.01). Specifically, IL-1β decreased by 57.99%, BUN decreased by 26.77%, MDA decreased by approximately 83.37%, and CRE decreased by 121.51%.

**Figure 5:**
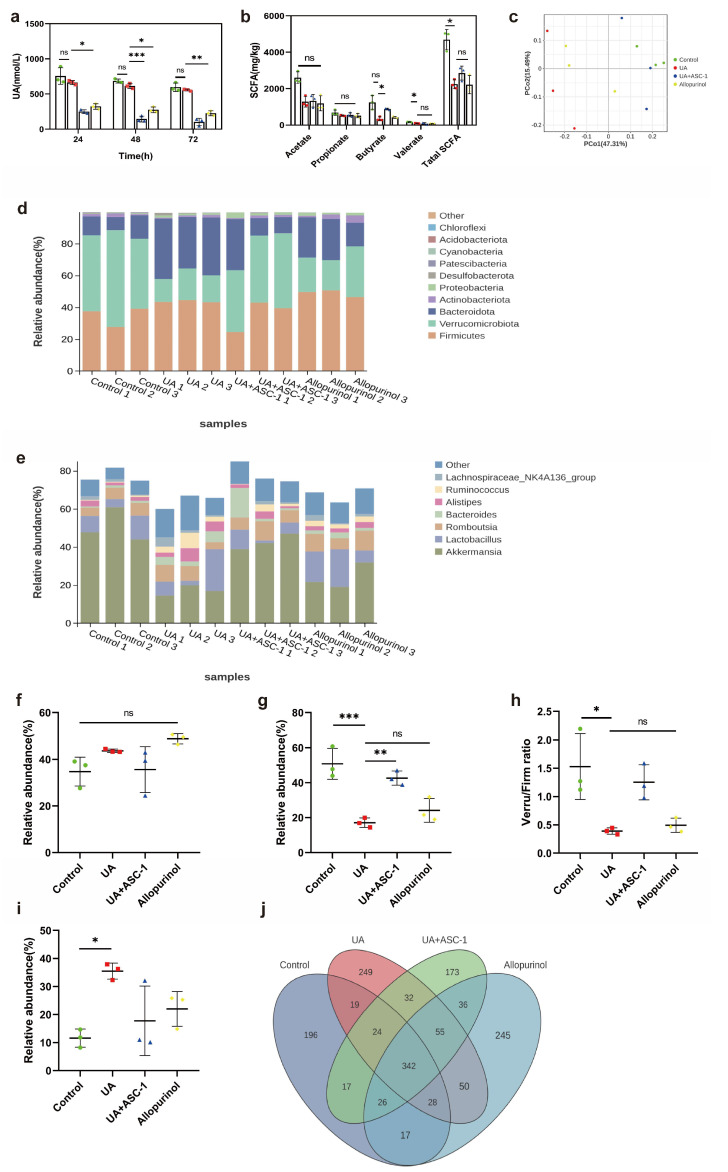
The impact of ASC-1 and allopurinol treatment on the functionality and diversity of the intestinal microbiota in rats exposed to UA. (**a**) The fecal microbiota from various groups of rats, indicating their ability to degrade UA. (**b**) The concentrations of short-chain fatty acids in the feces of these groups. (**c**) Principal Coordinate Analysis (PCoA). (**d**) Comparison of relative abundances at the phylum level among different groups. (**e**) Comparison of relative abundances of the most abundant bacterial genera among the four groups. (**f**,**g**,**i**) Comparison of relative abundances of significantly changed bacterial phyla (*Firmicutes*, *Verrucomicrob*, and *Bacteroidetes*). (**h**) Verru/Firm ratio (the ratio of *Verrucomicrob* to *Firmicutes*) for each group. (**j**) Analysis of the overlap of microbial OTUs (operational taxonomic units) among different groups using Venn diagrams. Bar graphs show the mean ± standard deviation of data from 3 rats in each group. * *p* < 0.1; ** *p* < 0.01; *** *p* < 0.001 indicate statistically significant differences.

In the UA-treated group, both kidney and liver tissues showed significantly elevated levels of IL-1β (*p* < 0.01). However, the ASC-1 + UA and allopurinol groups showed a decrease (Figure 4e,g). Specifically, the levels of IL-1β in the kidney and liver decreased by 75.40% and 60.30%, respectively. Moreover, the level of XOD increased by 58.87% in the livers of rats in the UA group but returned to the control group’s levels following ASC-1 intervention (Figure 4f). There was no significant difference in MDA content between the UA and ASC-1 groups in the kidneys of rats. However, MDA levels were significantly reduced in the allopurinol group (Figure 4h). Histopathological analysis showed that exposure to a high-purine diet and oxonic acid potassium resulted in liver injury, as evidenced by increased intercellular spaces (black arrows) and deeper nuclear staining (red arrows) in the liver sections from UA-treated rats compared to in those from control rats. These symptoms were partially alleviated in rats in the ASC-1 + UA and allopurinol groups (Figure 5i). However, no significant differences were observed between the UA + ASC-1 and allopurinol groups in these indicators. On the other hand, no significant differences were observed between the groups in terms of kidney tissue. These results further confirm the mitigating effects of ASC-1 on hyperuricemia.

### 3.4. ASC-1 Regulated UA-Induced Gut Microbiota Dysbiosis in Hyperuricemic Rats

To evaluate the impact of ASC-1 on the UA-degrading capacity of gut microbiota, the UA degradation capacity of fecal microorganisms from rats was compared before and after oral administration with ASC-1. The results showed that over 60% UA content was degraded by fecal microorganisms of the rats in the ASC-1 group (*p* < 0.1, Figure 5a). As shown in Figure 5b, rats in the UA group had significantly lower levels of total short-chain fatty acids (SCFAs) and valerate in their feces than rats in the control group (*p* < 0.1). On the contrary, the levels of butyric acid and SCFA were restored after oral administration of ASC-1 (*p* < 0.1, Figure 5b).

To further investigate the effect of ASC-1 on gut microbiota, the changes in the diversity and abundance of gut microbiota associated with UA exposure and/or treatment with ASC-1 were analyzed using Miseq sequencing technology. After the treatment, microbial diversity, as measured by the Shannon index, was higher in the UA + ASC-1 group than in the UA group. This result suggests that ASC-1 treatment positively affected microbial diversity. Our findings are similar to those of another study, in which dietary supplementation with Lactobacillus plantarum TCI227 slightly ameliorated the decline in microbial diversity [37]. Furthermore, the results of the Principal Coordinates Analysis (PCoA) showed significant differences in the phylogenetic community structure between the high-purine diet and oxonic acid potassium-exposed samples and the other samples, with the UA group being distinct from the other groups. These data suggest that UA exposure results in changes in the composition of the gut microbiota. However, the effect of UA on changes in microbial composition was attenuated by ASC-1 supplementation (Figure 5c).

The relative abundance at the phylum level for each sample in the four groups was further analyzed, as shown in Figure 5d. The dominant phyla in the gut microbiota were *Firmicutes*, *Verrucomicrobia*, *Bacteroidetes*, *Proteobacteria*, and *Actinobacteriota* (relative abundance > 0.5%). A significant decrease in the relative abundance of *Verrucomicrobia* and an increase in the relative abundance of *Bacteroidetes* were observed when comparing the UA and control groups. The impact of ASC-1 treatment on UA-induced changes in the abundance of *Firmicutes*, *Verrucomicrobia*, and *Bacteroidetes* is shown in Figure 5f,g,i. The Verru/Firm ratio decreased under UA exposure, but ASC-1 treatment improved this effect (Figure 5h).

At the genus level (Figure 5e), the results indicate that exposure to UA decreased the relative abundance of *Akkermansia* and of *Bacteroides*. These changes in the gut microbiota composition induced by UA were counteracted by ASC-1 treatment. *Akkermansia* levels in the UA + ASC-1 group were 249.88% higher than those in the UA group. This genus is typically observed in healthy individuals. Venn analysis further demonstrated that a high-purine diet and oxonic acid potassium treatment significantly affected the gut microbiota structure (Figure 5j). These results suggest a positive impact of ASC-1 on the gut microbiota.

## 4. Discussion

Hyperuricemia is a common metabolic disorder [3,4]. Its prevalence can be affected by excessive intake of high purine and high fructose foods [6,14]. Previous research has shown that hyperuricemia is more prevalent in coastal areas due to excessive purine intake [38,39,40]. To reduce blood uric acid levels, it is important to control the diet and intake of low-purine foods.

### 4.1. ASC-1 Efficiently Degraded Uric Acid and Inosine

Microorganisms often use precursors such as UA and nucleic acids as nutrients for growth. Therefore, some microorganisms exhibit uric acid degradation [13,41]. In this study, we found that the ASC-1 strain, isolated from pickled cabbage, showed good UA-degrading ability. In an in vitro assay, strain ASC-1 was able to degrade 77.14% of the total UA within 36 h and completely degrade inosine at a concentration of 1.26 mmol/L within 30 min. Furthermore, qPCR analysis demonstrated successful survival and colonization of ASC-1 in the intestines of rats during drug administration. Notably, ASC-1 significantly reduced serum UA (67.24%) in hyperuricemic rats. Additionally, *Limosilactobacillus fermentum* NCU003018 has the ability to degrade UA with an efficiency of 30.77% [42]. While *Bacillus paramycoides*-YC02 was found to completely degrade UA at a concentration of 500 mg/L within 48 h [43]. Inosine is a purine nucleoside metabolized to uric acid, and its degradation can indirectly affect uric acid levels. ASC-1 rapidly degrades inosine. Consistent with previous reports, *Lactobacillus paracasei* X11 was able to degrade both inosine and UA [44]. In summary, *B. megaterium* ASC-1 exhibits a strong ability to degrade UA and inosine, making it a promising candidate for the treatment of hyperuricemia. This study focused solely on ASC-1, which was the most efficient strain identified. However, it is noteworthy that the other two strains merit further exploration.

### 4.2. ASC-1 Alleviated Inflammation and Oxidative Stress in Hyperuricemia Rats

Hyperuricemia often leads to oxidative damage and inflammation due to UA, which can also cause liver and kidney damage [45]. IL-1β is a key pro-inflammatory factor, and high levels of uric acid promote the release of IL-1β and inflammatory responses. It is important to note that hyperuricemia can have various phenotypes [46,47]. This study shows that ASC-1 can effectively reduce MDA and IL-1β levels, effectively degrade nucleosides in the intestine, decrease inflammation, and ultimately alleviate liver and kidney tissue damage. Similar studies have shown that *Lacticaseibacillus rhamnosus* 1155 and *Limosilactobacillus fermentum* 2644 can effectively reduce the levels of inflammation and oxidative damage in rats with hyperuricemia [48]. It was discovered that *Lactobacillus fermentum* F40-4 alleviated the level of liver and kidney damage in hyperuricemia [49]. These results suggest that ASC-1 can relieve the inflammation and oxidative stress induced by hyperuricemia.

### 4.3. ASC-1 Suppressed Elevated XOD Activity in the Livers of Hyperuricemia Rats

Xanthine oxidoreductase (XOD) is an enzyme found primarily in the liver that catalyzes UA biosynthesis. It has been confirmed that excessive production of XOD leads to increased UA production, which in turn induces oxidative stress and liver inflammation. Inhibition of XOD has long been considered as one of the main methods for treating hyperuricemia [50,51]. The elevated XOD activity in the liver returned to normal levels after intervention with ASC-1 and allopurinol, indicating that ASC-1 and allopurinol inhibited XOD, which helped control the production of uric acid. *Lactobacillus paracasei* X11, which was isolated from traditional fermented foods, significantly reduced liver XOD activity in hyperuricemic mice induced by a high-purine diet by 33.69% [44]. The finding that *Lactobacillus brevis* DM9218 markedly inhibited liver XOD activity in high-fructose diet-induced hyperuricemic mice is consistent with this result [52]. Liver injury caused by a high-purine diet and oxonic acid potassium injection in rats was not severe, possibly due to the short modeling time. However, the injury still persisted. This may indicate that poor dietary habits or drug injections could potentially impact the liver, even for short periods of time under experimental conditions. Additionally, consistent with previous studies on LR1155 and LF2644, which were isolated from the Xinjiang and Inner Mongolia regions of China, intervention with ASC-1 significantly reduced the renal injury characteristics of hyperuricemia rats, as shown by H&E staining micrographs [48]. These results suggest that ASC-1 can reduce the XOD activity elevated by UA, which may be beneficial for alleviating hyperuricemia.

### 4.4. ASC-1 Restores the Gut Microbiota and SCFA Production in Hyperuricemia Rats

Short-chain fatty acids (SCFAs) are the primary end products of anaerobic bacterial metabolism in the colon. They are mainly produced by the metabolism of dietary fiber and other chemical water mixtures by beneficial intestinal bacteria. These acids play a crucial role in maintaining the normal function of the large intestine and the morphology and function of colon epithelial cells. Research has shown that short-chain fatty acids are associated with hyperuricemia and gout [9,53]. They can provide energy for intestinal epithelial cells to promote UA metabolism and relieve the inflammatory response caused by hyperuricemia and gout [37,54,55]. In this study, the role of ASC-1 in restoring normal gut microbiota metabolism was confirmed by an increase in the SCFA levels. In addition, in rats treated with a combination of UA and ASC-1 (UA + ASC-1 group), the fecal bacteria showed significantly greater UA degradation than rats treated with UA alone and allopurinol. This suggests that the oral administration of ASC-1 enhanced the ability of the gut microbiota in degrading UA. Therefore, ASC-1 may regulate intestinal metabolic status and alleviate gout symptoms or inflammatory responses by promoting UA degradation in the gut and restoring normal butyrate production. Based on the different regulatory mechanisms of allopurinol and ASC-1 in hyperuricemic rats, SCFA levels were not significantly increased in the allopurinol group.

Uric acid has a significant effect on the gut microbiota, as it acts as an antioxidant and immune enhancer [10]. In this study, UA exposure significantly reduced gut microbial diversity and abundance compared to that in the control group. At the phylum level, the relative abundance of *Firmicutes* significantly increased due to UA exposure, while the abundances of *Bacteroidota* and *Verrucomicrobiota*, which play key host functions such as metabolism, development, and immune properties, decreased. *Firmicutes* are often associated with energy metabolism and fat accumulation. An increase in their abundance may indicate a disruption in host energy metabolism [56,57,58]. These findings are similar to those of previous studies on hyperuricemic Sprague-Dawley rats [48]. However, pretreatment with ASC-1 significantly reduced the UA-induced increase in *Verrucomicrobiota* and decreased *Firmicutes* and *Bacteroidota*. ASC-1 may alleviate the symptoms of related diseases such as hyperuricemia by affecting the metabolic activities of the gut microbiota.

At the genus level, UA significantly reduced the abundance of *Akkermansia muciniphila* and increased the abundance of *Bacteroides*. *Akkermansia muciniphila* is a normal flora of the human intestine, and many studies have shown that its abundance is negatively correlated with obesity, diabetes, and cardiovascular disease [59,60]. When the abundance of *Akkermansia muciniphila* decreases, the host may be at higher risk of disease. Some scholars have pointed out that *Akkermansia muciniphila* is expected to be the next generation of probiotics after *Lactobacillus* and *Bifidobacterium* [61,62]. In this study, we observed a significant increase in *Akkermansia muciniphila* in ASC-1-fed rats, whereas a significant increase in *Lactobacillus* abundance was observed in rats treated with allopurinol. Therefore, increasing the abundance of *Akkermansia muciniphila* may improve the metabolic status of the host and prevent or alleviate the symptoms of related diseases. Consistent with our report, the addition of LR1155 and LF2644 supplements confirmed that the gut microbiota of rats was primarily dominated by *Firmicutes* [48]. Therefore, it can be concluded that dietary supplementation with ASC-1 can reduce hyperuricemia-associated phylum-level disturbances and maintain the ability of gut microbiota to degrade UA in hyperuricemic rats. Although the results of animal and human studies may differ, further studies are necessary to assess whether ASC-1 exerts similar beneficial effects in humans and to develop effective treatment strategies for hyperuricemia.

## 5. Conclusions

To summarize, in this study, a strain of *P. megaterium* ASC-1 was isolated from pickled cabbage. ASC-1 grew best in UA medium and had high efficiency for degradation of UA and inosine. ASC-1 can reduce UA levels and alleviate inflammatory responses, as well as liver and kidney damage in hyperuricemic rats. Furthermore, ASC-1 has been shown to restore gut flora diversity, promote the growth of beneficial bacteria, and increase SCFA levels. Although the mechanism underlying uric acid reduction requires further research, our findings suggest that ASC-1 holds promise as an adjuvant therapy for patients with hyperuricemia.

## Data Availability

The raw data supporting the conclusions of this article will be made available by the authors upon request.
